# Structure characteristics and combustion kinetics of the co-pyrolytic char of rice straw and coal gangue

**DOI:** 10.1038/s41598-024-67378-y

**Published:** 2024-07-15

**Authors:** Chunyan Xu, Chengjia Luo, Jun Du, Lang Liu, Jingjing Wang, Chenhong Yuan, Junjiang Guo

**Affiliations:** 1 School of Materials and Environment, Guangxi Minzu University, Nanning, 530006 Guangxi China; 2 Guangxi Key Laboratory of Advanced Structural Materials and Carbon Neutrality, Nanning, 530006 Guangxi China; 3 Guangxi Colleges and Universities Key Laboratory of Environmental-Friendly Materials and Ecological Remediation, Nanning, 530006 Guangxi China; 4 Chemical Engineering Institute, Guizhou Institute of Technology, Guiyang, 550003 Guizhou China

**Keywords:** Coal gangue, Co-pyrolytic char, Char characteristics, Combustion kinetics, Biofuels, Chemical engineering

## Abstract

Co-combustion is a technology that enables the simultaneous and efficient utilization of biomass and coal gangue (CG). Nevertheless, the factors that affect the combustibility of co-pyrolytic char, which represents the rate-determining step of the entire co-combustion process, remain unclear. This study investigates the impact of the physicochemical properties of co-pyrolytic char, including pore structure, carbon structure, and alkali metals, on the combustion characteristics. The TGA analysis indicates that the ignition and burnout temperatures of the co-pyrolytic char increase as the CG mixing ratio increases, resulting in a prolonged combustion. This is due to the fact that the carbon structure of the co-pyrolytic char becomes increasingly aromatic, accompanied by a reduction in aliphatic hydrocarbons and oxygen-containing groups as the CG mixing ratio increases. Furthermore, the high ash content of the CG is another significant factor contributing to the observed reduction in combustibility. The reaction between mullite, quartz in CG, and alkali metals in biomass results in the formation of aluminosilicate, which reduces the catalytic ability of alkali metals. Furthermore, the char combustion kinetics are analyzed by the KAS method, and the results indicate that the introduction of CG increases the activation energy of the entire char combustion process. The activation energy of the 80RS20CG is within the range of 102.22–164.99 kJ/mol, while the RS char is within the range of 89.87–144.67 kJ/mol.

## Introduction

In light of the dearth of fossil fuels and the imminent challenge of environmental contamination, it is imperative to investigate and develop clean and sustainable alternative sources of energy. Biomass resources are regarded as a prospective clean energy alternative to traditional fossil energy sources, given their abundance, renewability, storability and carbon neutrality^1–3^. Moreover, as a significant agricultural nation, China produces approximately 7.4 million tons of straw biomass annually^4^. The utilization of straw biomass has the potential to offer significant environmental and economic benefits. Thermal conversion technologies, such as pyrolysis, gasification, and combustion, are highly efficient methods for converting biomass into high-value fuel and/or electricity. In these processes, combustion accounts for approximately 97%^5–8^. Nevertheless, biomass typically contains a significant amount of alkali metals, which could result in a number of ash-related issues, such as ash melting, agglomeration, and corrosion, and thus reducing combustion efficiency^9–11^. Meanwhile, coal gangue (CG) is a by-product of the coal mining process^12^, and our previous research has demonstrated that the combination of mullite and quartz in CG can react with the alkali metals present in biomass during the combustion to form aluminosilicate with a high melting point^13^, helping to alleviate ash issues.

In addition, CG is a low calorific mixture composed of various minerals that is commonly used as a fuel for power generation, but is difficult to ignite and unstable in combustion. While biomass has a high volatile matter content, low ash content, and low ignition temperature^14,15^, making it favorable for improving the combustibility of CG^16^. Bi et al.^17^ demonstrated that the addition of peanut shells can enhance the combustibility of CG and increase gas generation during co-combustion. Zhang et al.^18^ found that the inclusion of pine sawdust has a positive impact on CG combustion. Bi et al.^19^ investigated the co-combustion kinetics of CG and peanut shell by the artificial neural network method. Their findings indicated the activation energy of the co-combustion of CG and peanut shell was significantly lower than that of CG combustion alone. Li et al.^20^ demonstrated that the volatile matter precipitated from polypropylene can significantly reduce the activation energy during the initial stage of co-combustion of CG and polypropylene. In conclusion, co-combustion is expected to alleviate the ash problem caused by alkali metals in biomass^13^ and enhance the combustion efficiency^21–24^, thereby optimizing the utilization of both solid wastes.

Nevertheless, the majority of current studies focus on the kinetics, ash issues and gas generation during the co-combustion, with few scholars paying attention to the combustion characteristics of the co-pyrolytic char, which is the rate-determining step of the entire co-combustion process^25,26^. The combustibility of the co-pyrolytic char is directly correlated with its physicochemical properties, including pore structure, carbon structure and inorganic minerals. Tilghman et al.^27^ demonstrated that alterations in the carbon structure of biochar result in changes in its gasification and combustion reactivity. Tong et al.^28^ confirmed that the chemical structure is a primary determinant of the biochar activity. Zou et al.^26^ demonstrated that the combustion of the biochar is related to the number of the aromatic rings. Meanwhile, the alkali metals present in biomass have the potential to enhance the combustion activity of the biochar^29^. In summary, the char combustion characteristic is directly related to its physicochemical properties, including pore structure, carbon structure and the presence of alkali metals. Moreover, studies have indicated that the minerals present in CG can facilitate the formation of carbon during the biomass pyrolysis^30^, resulting in the generation of char with a developed pore structure and stable aliphatic chain structure^31^. Nevertheless, the precise mechanism by which the introduction of CG affects the physicochemical properties of co-pyrolytic char, and the relationship between these physicochemical properties and the combustibility remain unclear.

The objective of this study is to investigate the physicochemical properties of co-pyrolytic char produced with varying biomass and CG mixing ratios, to characterize its combustion behavior, and to examine the influence of the physicochemical properties on the combustion kinetics. The results will provide valuable insights into the interaction between biomass and CG during co-combustion process.

## Material and methods

### Raw materials

The CG used in this study was sourced from a coal mine in southwestern China, while the rice straw was obtained from rural areas in southern China. Both samples were ground and sieved into particles with a diameter of less than 74 μm, and subsequently air-dried in an oven at 105 °C for 12 h. The proximate, ultimate and heating value analysis of the CG and RS are shown in Table 1. The fixed carbon content in RS and CG is comparable, as evidenced by the proximate analysis. While RS exhibits a markedly higher volatile matter content and a significantly lower ash content than CG. The inorganic mineral compositions in RS and CG are presented in Table 2. It shows that CG mainly contains 52.89% SiO_2_, 22.32% Al_2_O_3_, and 10.12% Fe_2_O_3_, while RS mainly contains 44.16% SiO_2_ and 18.89% K_2_O.

**Table 1:** Proximate and ultimate analysis and heating value of RS and CG used in this study.

Properties	Sample	
	RS	CG
Proximate analysis, ad (wt.%)		
Fixed carbon	15.22	16.23
Volatile matter	78.64	9.61
Ash	6.14	74.16
Ultimate analysis, ad (wt.%)		
C	44.02	17.49
H	4.83	1.18
N	1.72	0.40
O^b^	43.01	4.49
S	0.28	2.28
H/C	1.32	0.81
O/C	0.73	0.19
HHV (MJ/kg)	16.45	5.69

**Table 2:** Inorganic minerals composition analysis in ash of CG and RS.

Composition (%)	Sample	
	RS	CG
SiO_2_	44.16	52.89
Al_2_O_3_	3.16	22.32
Fe_2_O_3_	0.61	10.12
CaO	7.62	3.86
Cl	6.35	0.12
K_2_O	18.89	3.12
SO_3_	3.89	2.56
Na_2_O	3.53	2.32
MgO	6.88	1.72
P_2_O_5_	2.81	0.48
MnO	1.72	0.21

### Co-pyrolysis experiment

**Figure 1:**
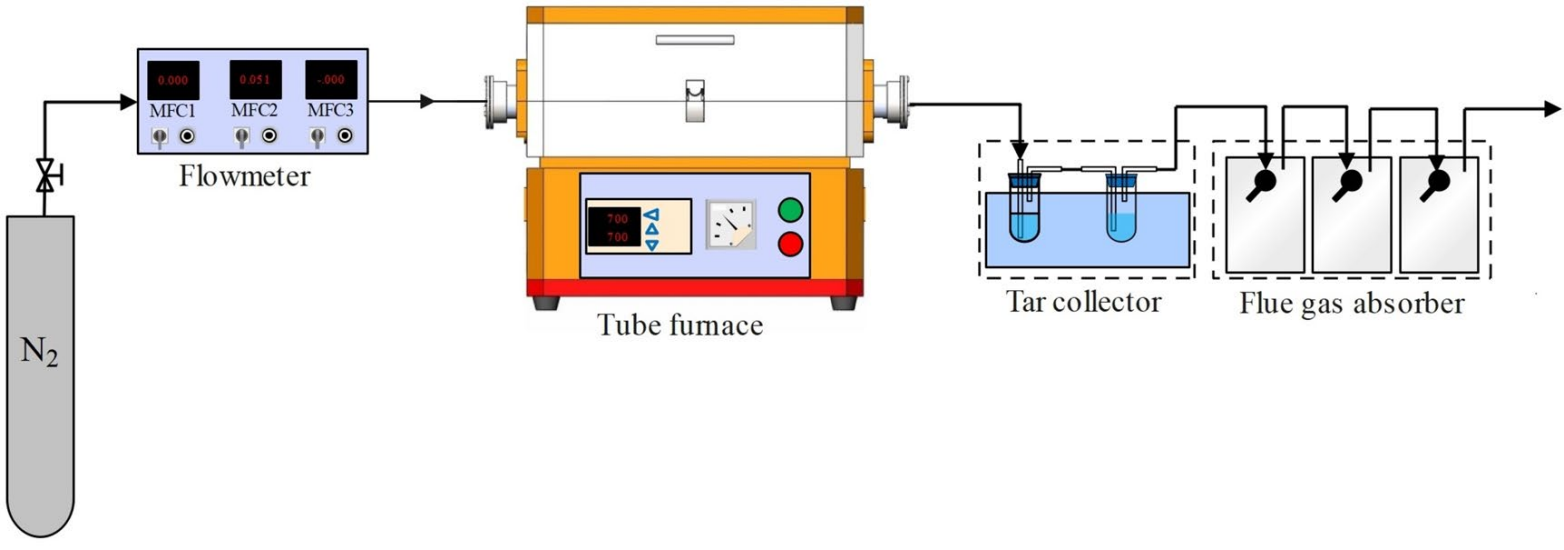
The schematic diagram of the co-pyrolytic experimental setup.

Six feedstocks were obtained by mixing RS and CG in the mass ratios of 100:0, 80:20, 60:40, 40:60, 20:80 and 0:100, respectively. In the co-pyrolysis experiment, 5 g of the feedstocks was spread in an Al_2_O_3_ crucible and heated up to 500 °C in a tube furnace at a rate of 20 °C/min and held for 30 min under an N_2_ atmosphere with a purity of 99.9% by mole. The generated gas was extracted from the tube furnace with N_2_ and condensed in two scrubbers immersed in a water bath to collect the generated tar, which was then weighed. The residual char samples were collected and weighed after the furnace had cooled down under an N_2_ atmosphere, and named according to the ratio of RS to CG in the feedstocks: RS char, 80RS20CG, 60RS40CG, 40RS60CG, 20RS80CG, and CG char. The schematic diagram of the co-pyrolysis experimental setup is shown in Fig. 1, and all experiments were repeated three times to obtain an average and minimize errors.

### Physicochemical characteristics of co-pyrolytic char samples

Various analytical techniques and instruments were used to determine the physicochemical properties of the co-pyrolytic char samples. The pore structure parameters of the co-pyrolytic char samples were determined using an N_2_ adsorption technique in a BET apparatus (Micromeritics ASAP 2420). The microstructures were observed using a SEM (ZEISS EVO 18). The organic functional groups were characterized using a FTIR spectrometer (NicoletiS50, Thermofisher Scientific). The carbon structures were analyzed using a Raman spectrometer (Thermo Fischer DXR), and the mineral compositions were determined using XRD (Ultima IV).

### Combustion characteristics of the co-pyrolytic char

The combustion experiment of the co-pyrolytic char samples was carried out on a thermogravimetric analyzer (TGA 550, TA). In each experiment, approximately 5 mg char was placed in an Al_2_O_3_ crucible, which was then heated to 973.15 K at different heating rates in an air atmosphere with a flow rate of 50 mL/min. Meanwhile, the weight losses during the combustion were converted into combustion conversion rates to allow a comparative analysis of the combustion characteristics of the co-pyrolytic char samples, and the combustion conversion was calculated via the Eq. (1):

1 $$ x = \frac{{w_{0} - w_{t} }}{{w_{0} - w_{\infty } }} \times 100\% $$

The reaction rate (*dx*/*dt*) was calculated by analyzing the combustion conversion rate versus time profiles using Eq. (2).

2 $$ Reaction\;rate = \frac{dx}{{dt}} $$

where *w*_0_, *w*_*t*_, and *w*_*∞*_ denote the initial weight, the weight at the time *t* and the final weight of the co-pyrolytic char samples during the combustion, respectively.

## Results and discussion

### The mass yields of char, tar and gas during the co-pyrolysis

**Figure 2:**
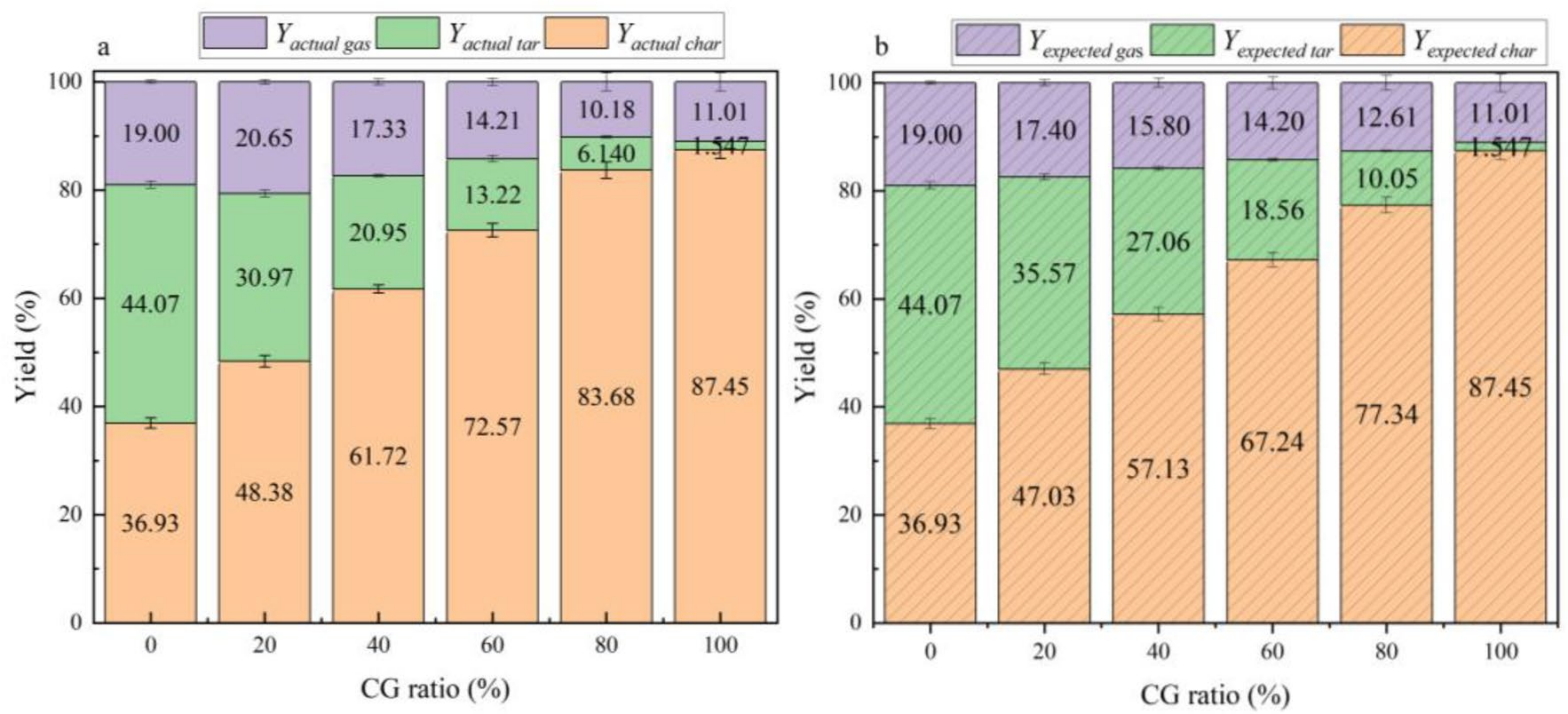
The actual & expected mass yields of the char, tar and gas during pyrolysis.

**Figure 3:**
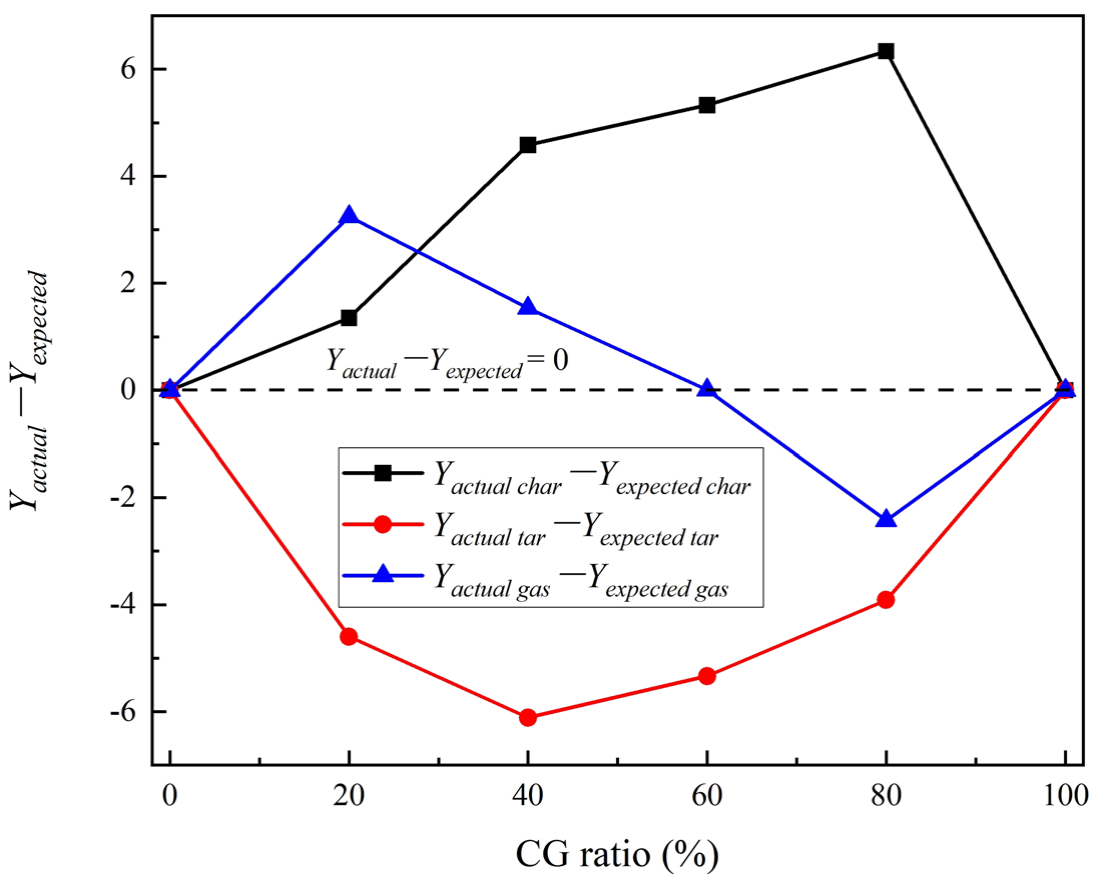
Difference between actual and expected mass yields.

According to the weight of the generated tar and residual char measured in the pyrolysis experiment, the actual mass yields of char, tar and syngas, named as *Y*_*actual char*_, *Y*_*actual tar*_ and *Y*_*actual gas*_, respectively, can be calculated by Eqs. (3–5). Furthermore, the expected mass yields of char, tar and syngas, named as* Y*_*expected char*_, *Y*_*expected tar*_ and *Y*_*expected gas*_, respectively, based on the assumption that there is no interaction between CG and RS during the pyrolysis are introduced to analyze the influence of CG on RS pyrolysis. The expected mass yields are estimated by Eqs. (6–8).

3 $$ Y_{actual\;char} = W_{char} /W_{0} \times 100\% $$

4 $$ Y_{ actual\;tar} = W_{tar} /W_{0} \times 100\% $$

5 $$ Y_{actual\;gas} = 100\% - Y_{actual\;char} - Y_{actual\;tar} $$

6 $$ Y_{expected\;char} = Y_{RS\;char} \times x_{RS} + Y_{CG\;char} \times x_{CG} $$

7 $$ Y_{expected\;tar} = Y_{RS\;tar} \times x_{RS} + Y_{CG\;tar} \times x_{CG} $$

8 $$ Y_{expected\;gas} = 100\% - Y_{expected\;char} - Y_{expected\;tar} $$

where *W*_*0*_ is the initial weight of feedstock; *W*_*char*_ and *W*_*tar*_ are the actual weight of the residual char and generated tar during the pyrolysis, respectively; *Y*_*RS char*_ and *Y*_*RS tar*_ are the mass yields of the residual char and tar when RS was pyrolyzed separately, respectively; *Y*_*CG char*_ and *Y*_*CG tar*_ are the mass yields of char and tar when CG was pyrolyzed separately, respectively; *x*_*RS*_ and *x*_*RS*_ represent the mixing ratio of RS and CG in the feedstocks, respectively. The calculated actual mass yields and expected mass yields are given in Fig. 2, and the difference between actual and expected mass yields are presented in Fig. 3. It shows that the values of *Y*_*actual tar*_ − *Y*_*expected tar*_ < 0,* Y*_*actual char*_ − *Y*_*expected char*_ > 0 and *Y*_*actual gas*_ − *Y*_*expected gas*_ > 0 with a CG mixing ratio < 60%. As known, the pyrolysis under N_2_ atmosphere mainly involves drying, pyrolysis, and tar cracking, which sequentially undergoes R1 and R2 reactions, and the main products include the residual char, tar and syngas^7^. The volatile matter of the feedstock undergoes cracking to form tar and gas through R1, which is subsequently released, resulting in a decrease in the residual char yield. However, the actual tar yield is lower than the expected tar yield, and the actual char yield is greater than the expected char yield. This is mainly due to the dehydroxylation of kaolinite in CG during pyrolysis delays the residence time, thus accelerating the pyrolysis reaction and increasing the carbon yield^30^.

R1 $$ Pyrolysis:feedstock \to char + tar + gas $$

R2 $$ Cracking:tar \to {\text{C}} + {\text{CO}} + {\text{H}}_{2} + {\text{CH}}_{4} \ldots $$

As the CG mixing ratio increase to exceed 60%, the actual char mass yield is greater than the expected char mass yield, which is due to the accumulation of ash in CG hindering the pore structure, and a large number of organic components in RS accumulates on the surface of the ash, inhibiting the release of organic components and subsequent pyrolysis and cracking^30,32^.

R3 $$ Feedstock\;pyrolysis:feedstock \to char + tar + gas $$

R4 $$ Cracking:tar \to {\text{C}} + {\text{CO}} + {\text{H}}_{2} + {\text{CH}}_{4} \ldots \, $$

### Pore structure of the co-pyrolytic char

**Figure 4:**
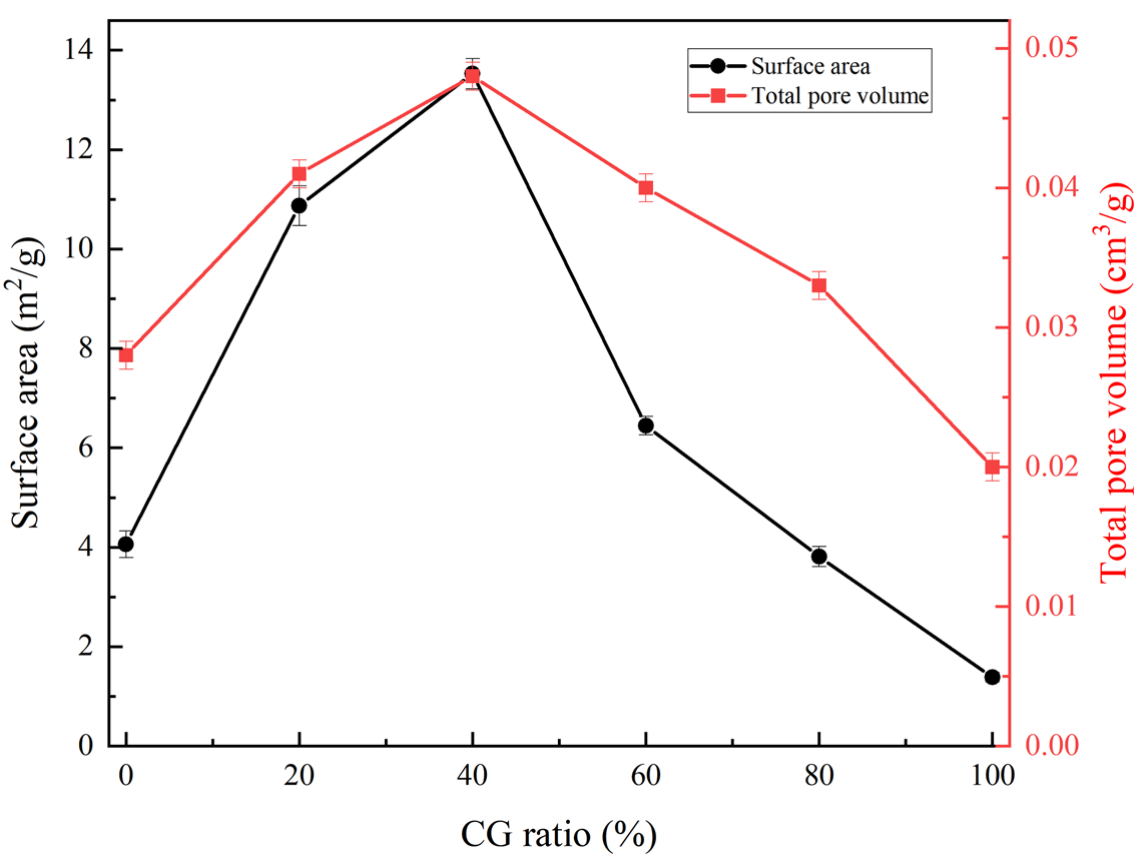
Specific surface area and total pore volume of the co-pyrolytic char samples.

The pore structural parameters of the co-pyrolytic char samples, including total pore volume and specific surface area, were quantitatively characterized using the N_2_ adsorption isotherms method, and the results are shown in Fig. 4. Figure 4 shows that both the total pore volume and the specific surface area increased gradually as the CG mixing ratio increased from 0 to 40%. This is mainly because CG is conducive to catalyzing the pyrolysis of volatile matter in RS into tar, carbon and gas. The formation of tar and syngas can result in the emptying of the pores, while the formation of carbon can act as a skeleton to support these pores, thereby favoring the development of pore structure. As the CG mixing ratio increases from 40 to 100%, the amount of inorganic minerals introduced also increases, which results in the co-pyrolytic char particles becoming more analogous in structure to rock, thereby reducing the pore structure.

**Figure 5:**
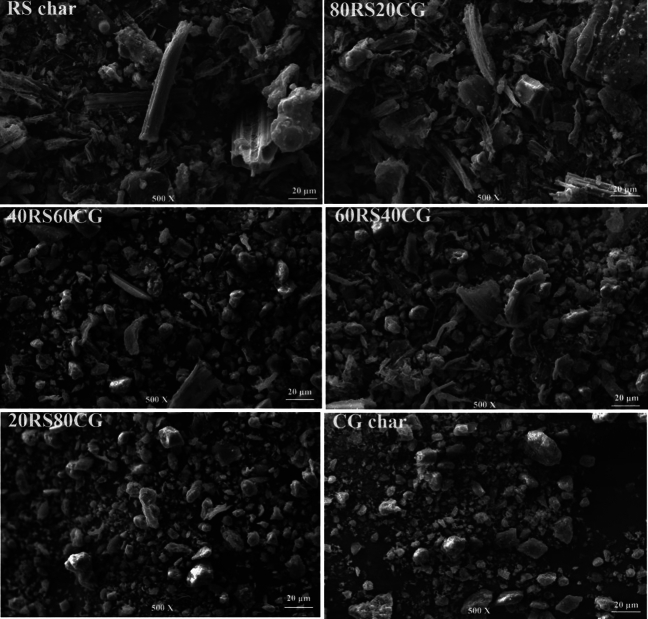
SEM images of the co-pyrolytic char samples.

In order to visualize the microstructure of the co-pyrolytic char samples, their micro-morphology was characterized by SEM. The results are shown in Fig. 5, and it shows that broken fiber structures and micro-sphere structures were observed on the surface of the particles in RS char, 80RS20CG and 60RS40CG. However, the number of these structures decreased gradually with an increase in the CG mixing ratio. The primary factors contributing to this phenomenon are that the CG is conducive to the pyrolysis of the cellulose into tar and gas, with the resulting tar undergoing further cracking into carbon and gas^30^. As the CG mixing ratio increases from 40 to 100%, the particles become more regular and smoother. This is due to the introduction of a large number of inorganic minerals by the CG, which results in the surface structure of the co-pyrolytic char particles becoming more similar to the rock structure with fewer dense pores. The rock structure with few dense pores would be expected to increase the resistance to heat and mass transfer, thereby inhibiting the co-pyrolysis of RS and CG.

### Chemical structure characteristics of the co-pyrolytic char

The proximate and ultimate analysis of the co-pyrolytic char are presented in Table 3. In comparison to the raw RS, the fixed carbon content in RS char exhibited a notable increase, rising from 15.22 to 55.24%. Conversely, the volatile matter content exhibited a significant decrease, falling from 78.64 to 26.07%. Additionally, the ash content demonstrated an upward trend, increasing from 6.14% to 18.69%. It is well established that the raw RS primarily comprises cellulose, hemicellulose, and lignin. The organic functional groups present in cellulose and hemicellulose undergo a process of breakage and recombination during pyrolysis, resulting in the formation of tar, CO_2_, CO, CH_4_, and H_2_, which in turn leads to a reduction in the volatile matter content^33^. Furthermore, the volatile matter in the RS also undergoes a slow carbon-forming reaction^6,34^. The reduction in the volatile matter content and carbon formation results in an increase in the fixed carbon content in RS char. The results also indicate that as the CG mixing ratio increases from 0 to 100%, the fixed carbon content in the co-pyrolytic char samples gradually decreases from 55.2% to 18.66%, the volatile matter content decreases from 26.07 to 5.22%, and the ash content increases from 18.69 to 76.12%. The contents of C, H, N and O in the char gradually decrease with the increase in CG mixing ratio. The above phenomena are mainly caused by the following two reasons: (1) the volatile matter in RS will break and recombine into tar, CO_2_, CO, CH_4_ and H_2_, and release under such co-pyrolysis condition^2,33^; (2) the addition of CG introduces a large amount of inorganic minerals.

**Table 3:** Proximate and ultimate analysis of the co-pyrolytic char samples.

Properties	Sample					
	RS char	80RS20CG	60RS40CG	40RS60CG	20RS80CG	CG char
Proximate analysis, ad (wt.%)						
Fixed carbon	55.24	43.28	30.58	22.96	19.81	18.66
V olatile matter	26.07	18.22	15.99	10.17	7.61	5.22
Ash	18.69	38.5	53.43	66.87	72.58	76.12
Ultimate analysis, ad (wt.%)						
C	64.42	49.36	36.91	25.56	21.56	19.32
H	2.54	1.99	1.54	1.38	1.27	1.12
N	1.41	0.83	0.57	0.39	0.31	0.24
O^b^	12.71	8.31	5.88	3.88	2.28	1.08
S	0.23	1.01	1.67	1.92	2.00	2.12
H/C molar ratio	0.47	0.48	0.50	0.65	0.71	0.70
O/C molar ratio	0.15	0.13	0.12	0.11	0.08	0.04

**Figure 6:**
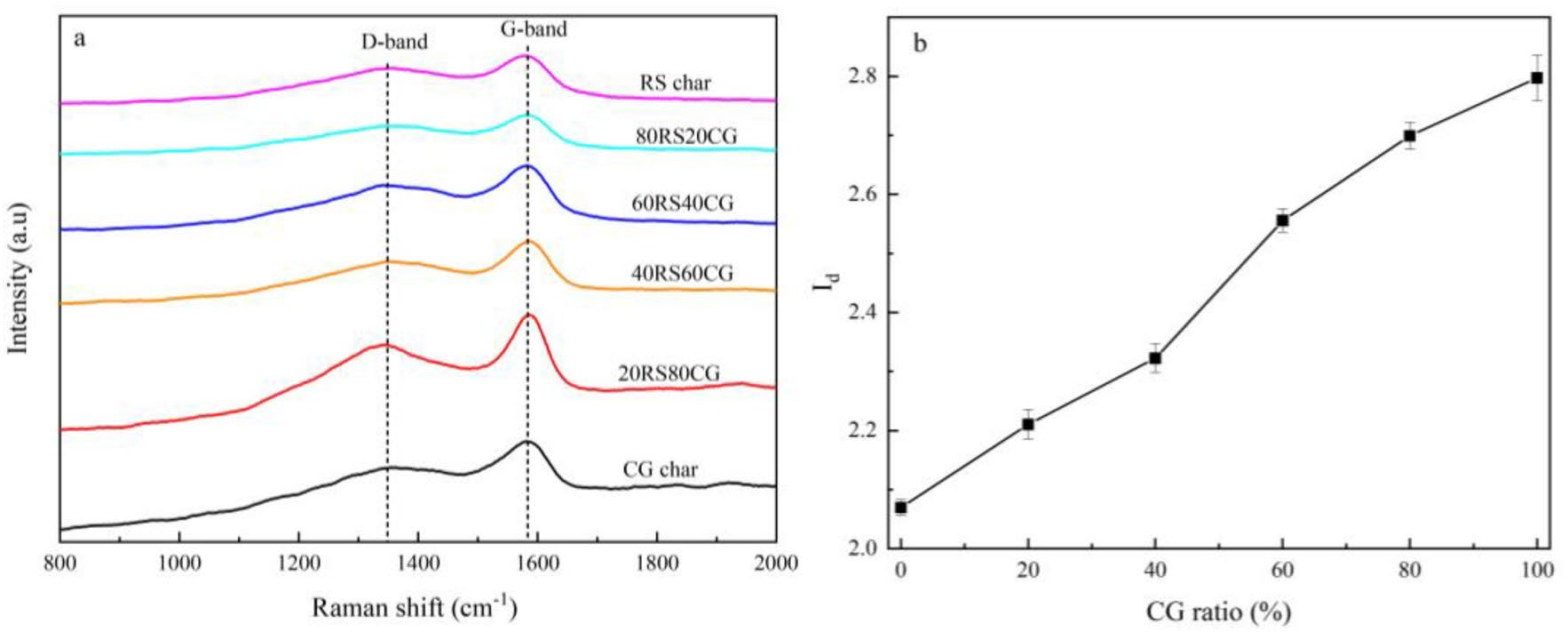
Raman spectra of the co-pyrolytic char samples.

The carbon structures of the different co-pyrolytic char samples were analyzed using the Raman spectroscopy, as shown in Fig. 6a. The Raman spectra of the samples can be roughly designated as a disordered carbon band (D band) at ~ 1380 cm^−1^ and a graphite band (G band) at ~ 1580 cm^−1^^35,36^. The results indicate that the Raman intensity of co-pyrolytic char exhibits a positive correlation with an increase in the CG mixing ratio increases. The introduction of greater quantities of metal oxides with an elevated of CG mixing ratio has the effect of reducing the light absorptivity, which in turn leads to an increase in the intensity of the Raman peaks. Moreover, the ratio *R*_*d*_, is a very important parameter to study the carbon structural information in co-pyrolytic char, and is defined as:

9 $$ R_{d} = \frac{{I_{d} + I_{g} }}{{I_{g} }} $$

where *I*_*d*_ and *I*_*g*_ are referred to the intensities of D-band and G-band, respectively, larger values of *R*_*d*_ indicate more disorder or amorphous carbon in the char^35,36^. The calculated *R*_*d*_ for each co-pyrolytic char sample is shown in Fig. 6b, which demonstrates that the value of *R*_*d*_ increases as the CG mixing ratio decreases. This suggests that the relative content of disordered or amorphous carbon in the co-pyrolytic char increases as the CG mixing ratio increases. This result is consistent with the ultimate analysis of the co-pyrolytic char samples presented in Table 3.

**Figure 7:**
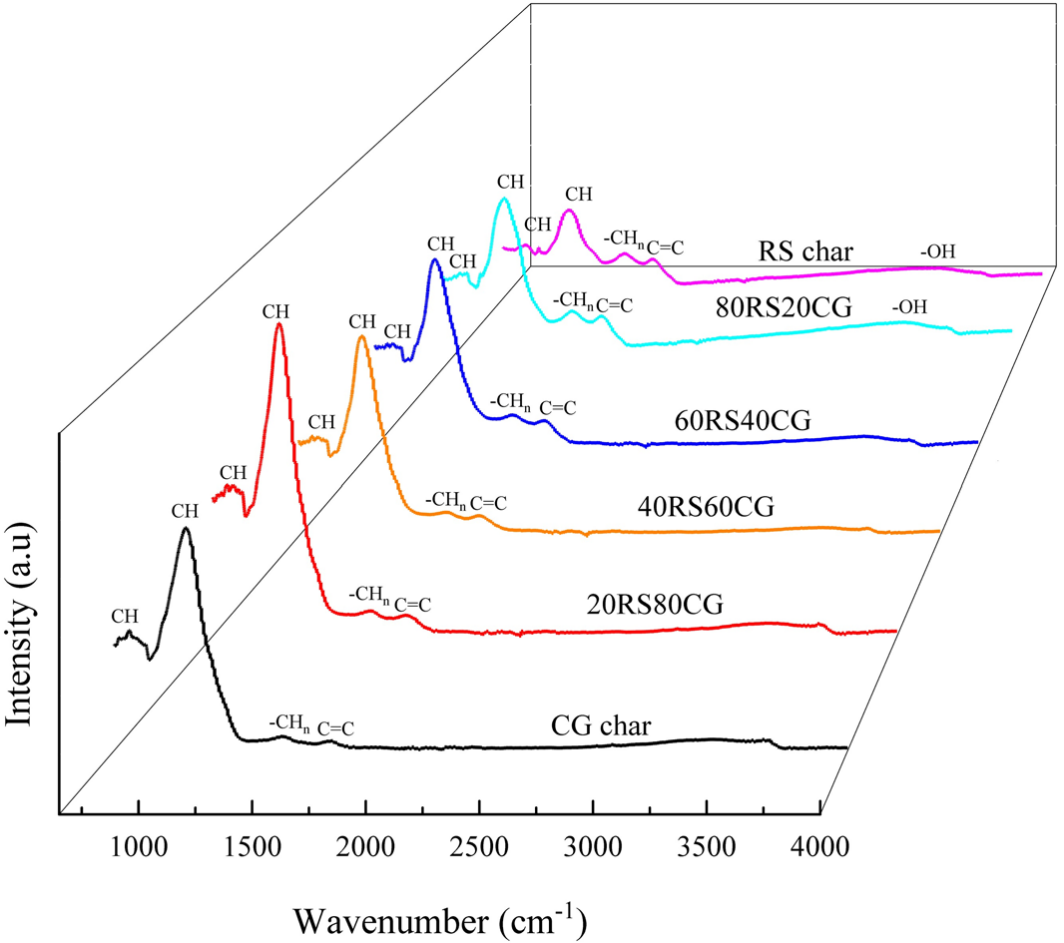
FTIR spectra of the co-pyrolytic char samples.

The FTIR spectra were employed to investigate the organic functional groups present in the co-pyrolytic char samples, as shown in Fig. 7. The results demonstrate that the groups present in the co-pyrolytic char samples are primarily comprised of the bending vibration bands of aromatic CH at ~ 780 cm^−1^ and ~ 1000 cm^−1^, the aliphatic -CH_n_ absorption bands at ~ 1410 cm^−1^, the aromatic C=C stretching vibration bands at ~ 1605 cm^−1^, as well as the –OH absorption bands in phenol groups at ~ 3300 cm^−1^^35,37^. The results also demonstrate that an increase in the CG mixing ratio leads to an enhancement in the peak strength of CH, while the peak strength of –OH, C=C, –CH_n_ and C–O bands is reduced. This is attributed to the organic functional groups in CG being predominantly aromatic groups, with the content of other organic functions decreasing as the CG mixing ratio increases. In addition, the inorganic components, such as Fe_2_O_3_, present in CG can facilitate the cracking of oxygen-containing functional groups in RS. This, in turn, results in a reduction in the content of the C–O and –OH bands^30^, thereby rendering the co-pyrolytic char more aromatic.

**Figure 8:**
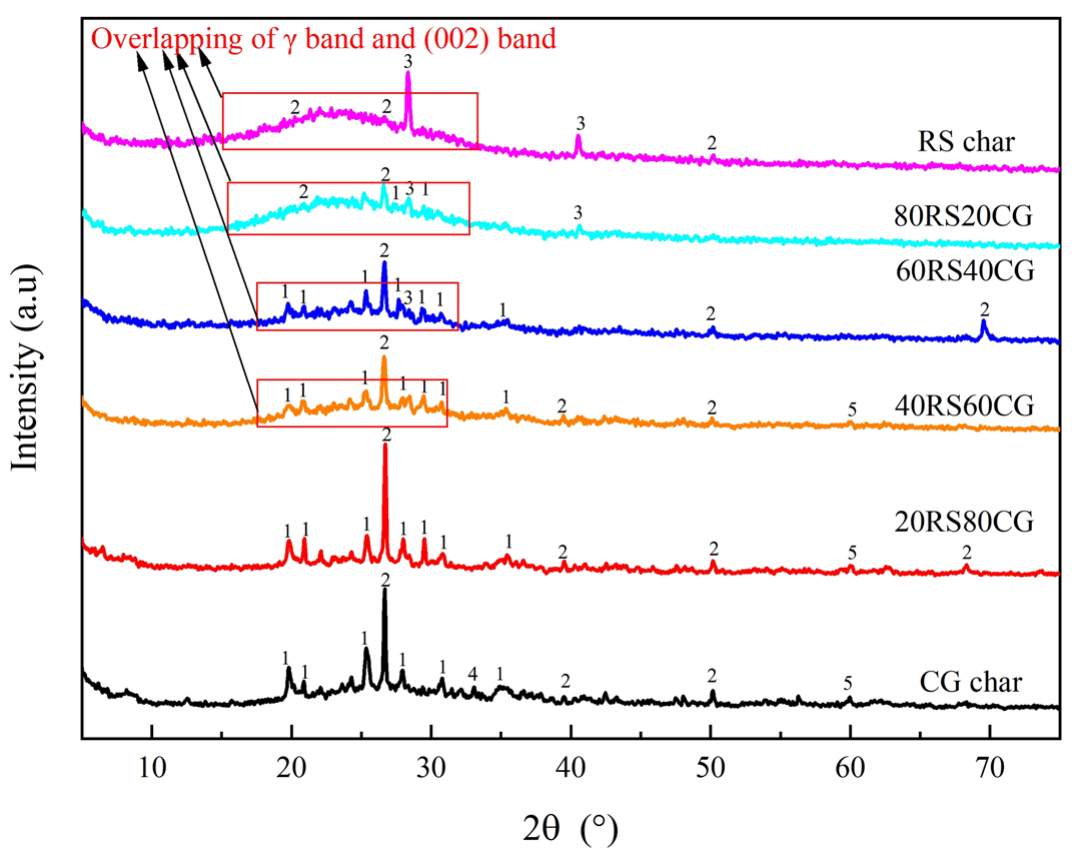
XRD patterns of the co-pyrolytic char samples. (1: Potassium mica (KAl_3_Si_3_O_11_); 2: Quartz (SiO_2_); 3: Sylvite (KCl); 4: Mullite (3Al_2_O_3_·2SiO_2_); 5: Alumina (Al_2_O_3_)).

The inorganic mineral composition and carbon structure of the char samples were determined by XRD, and the results are presented in Fig. 8. The results indicate that the primary minerals present in RS char are quartz and sylvite, while the main minerals in CG char are potassium mica, quartz, alumina and mullite. Whereas, there is no discernible mullite diffraction peak in the XRD curves of 80RS20CG, 60RS40CG, 40RS60CG and 20RS80CG. Furthermore, the sylvite diffraction peaks disappear as the CG mixing ratio increase to exceed 60%, indicating that the sylvite should react with mullite and quartz in CG to form potassium mica^13^. Nevertheless, potassium mica is a stable aluminosilicate with minimal catalytic effect on char combustion^38^. The broad bands observed in the range 2θ ≈ 15–26° are attributed to the overlap of the γ-bands, which indicates the presence of saturated aliphatic chains^39^. The asymmetric (002) bands observed in the range 2θ ≈ 15–32° reflect the degree of parallel orientation of the aromatic structural units^40^. Meanwhile, the background intensity of XRD spectrum is closely connected to the presence of amorphous carbon^41^. It can be observed that the background as well as the intensity of (002) bands and γ-bands gradually decrease in the CG mixing ratio, indicating that the carbon structure in the CG is minimal, which subsequently results in a reduction of amorphous and saturated aliphatic chains in the co-pyrolytic char as the CG mixing ratio increasing.

### Combustion characteristics of the co-pyrolytic char

**Figure 9:**
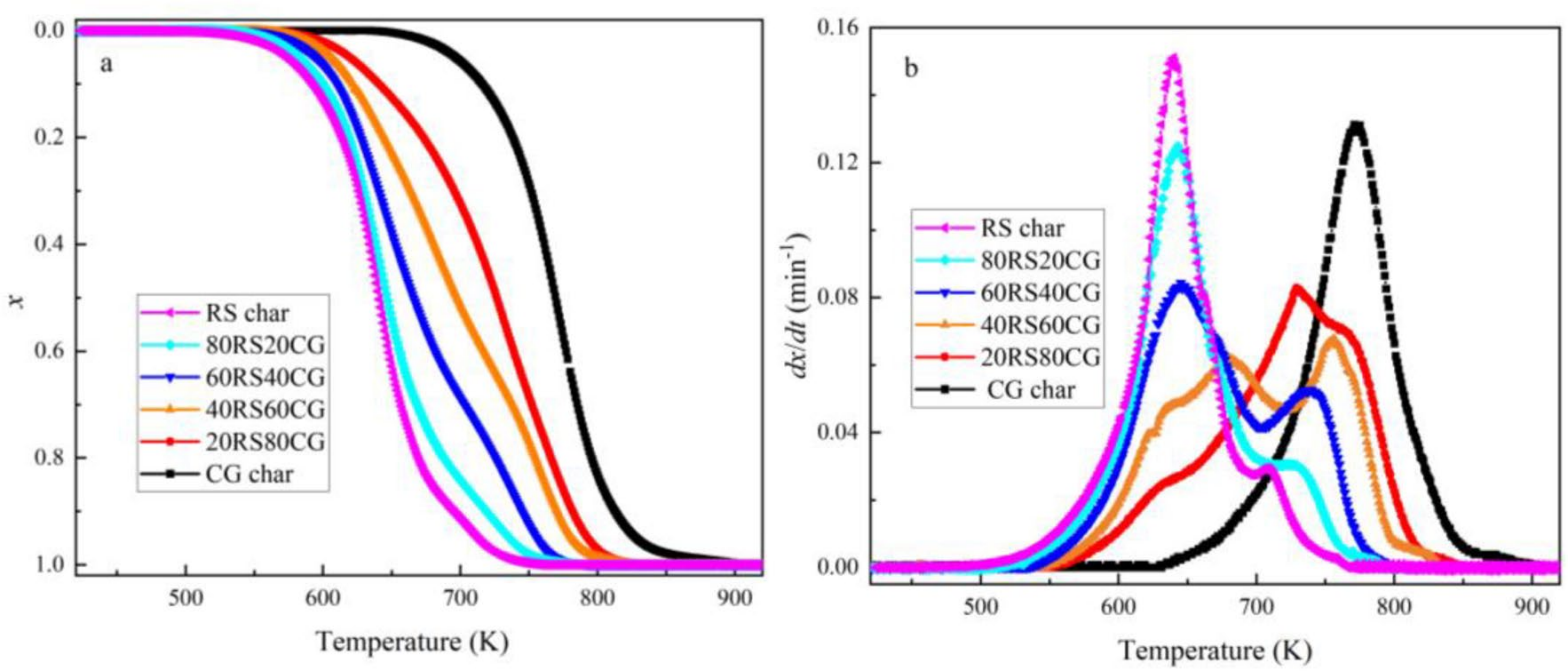
Combustion conversion rate (*x*) and reaction rate (*dx*/*dt*) curves of the co-pyrolytic char samples at 10 K/min under the air atmosphere.

The TGA was employed in this study to investigate the combustion characteristics of the co-pyrolytic char samples. The curves of combustion conversion rate and reaction rate for the co-pyrolytic char samples are presented in Fig. 9. Figure 9 illustrates that the reaction rate curves of the RS char, 80RS20CG, 60RS40CG, 40RS60CG and 20RS80CG exhibit two distinct peaks, suggesting that the combustion process can be divided into two primary stages. As the CG mixing ratio increased, both peaks shift to higher temperatures, accompanied by a decrease in the area of the low-temperature region and an increase in the area of the high-temperature region. As the CG mixing ratio increased to 100%, the CG char combustion primarily occurs between 632 and 912 K, with a single peak in the reaction rate curve observed at the higher temperature (761 K). The primary reason for this discrepancy is that the RS char contains a considerable number of aliphatic rings, oxygen-containing functional groups, and aromatic rings, whereas the functional group in CG char is primarily aromatic rings, as evidenced by the results of the FTIR spectra. In contrast, the oxygen-containing functional groups and aliphatic rings exhibit greater combustion reactivity than the aromatic rings^4^, which leads to the two distinct combustion regions. Furthermore, as the CG mixing ratio increased, the content of carbon structure in the co-pyrolytic char decreased significantly, while the relative content of aliphatic rings and oxygen-containing functional groups decreased more markedly than that of aromatic rings, as evidenced by the results of XRD, Raman and FTIR. This phenomenon results in the both combustion zones moving towards the higher temperature, and the area of the low-temperature combustion zone corresponding to aliphatic rings and oxygen-containing functional groups shows a gradual decrease, while the area of the high-temperature combustion zone corresponding to aliphatic rings exhibits a gradual increase shows a gradual increase. It should be noted that the increase in ash content impedes the contact between the gas and the active/available site, thereby reducing the combustion rate.

Meanwhile, the combustion parameters, including the initial temperature (*T*_*i*_) and burnout temperature (*T*_*f*_) were defined to evaluate the combustion parameters of the co-pyrolytic char samples^42^. The results are presented in Table 4, which demonstrates that the *T*_*i*_ value increases gradually with an increasing in the CG mixing ratio: 459.15 K (RS char) → 512.15 K (80RS20CG) → 536.15 K (60RS40CG) → 547.15 K (40RS60CG) → 552.15 K (20RS80CG) → 632.15 K (CG char). This suggests that the ignition temperature of the co-pyrolytic char increases with an increasing in the CG mixing ratio. A similar trend is observed in the burnout temperature, indicating that the co-pyrolytic char with a higher CG mixing ratio requires a higher temperature and longer time to burn out.

**Table 4:** Combustion characteristic parameters of the char samples.

Sample	*T*_*i*_ (K)	*T*_*m*−1_ (K)	*T*_*m*−2_ (K)	*T*_*f*_ (K)	*R*_max−1_ (min^−1^)	*R*_max*−*2_ (min^−1^)
RS char	459.15	640.15	708.15	766.15	0.15	0.0294
80RS20CG	512.15	643.15	723.15	792.15	0.126	0.0306
60RS40CG	536.15	645.15	740.15	797.15	0.084	0.0528
40RS60CG	547.15	680.15	756.15	836.15	0.06	0.066
20RS80CG	552.15	729.15	765.15	853.15	0.084	0.072
CG char	632.15	–	771.15	912.15	-	0.132

**Figure 10:**
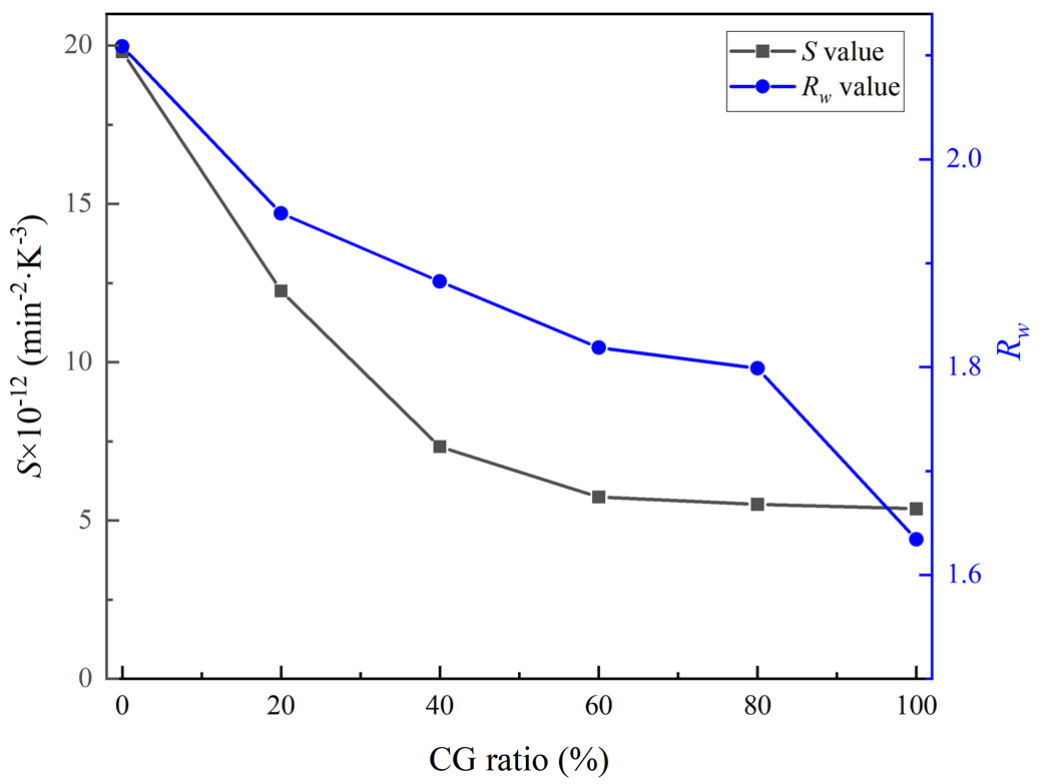
The calculated values of *S* and *R*_*w*_ of the co-pyrolytic char samples.

In addition, *S* and *R*_*w*_ related to ignition temperature, combustion rate, and burnout temperature were introduced to quantify the combustion reactivity and combustion stability of the char samples^22,43^. It should be noted that the larger *S* indicates the higher combustion reactivity, and the larger *R*_*w*_ means the more stable combustion, and the *S* and *R*_*w*_ can be described as^43,44^:

10 $$ S = \frac{{(dx/dt)_{\max } \times (dx/dt)_{mean} }}{{T_{i}^{2} \times T_{f} }} $$

11 $$ R_{w} = \frac{560}{{T_{i} }} + \frac{650}{{T_{\max } }} + 0.27 \times (dx/dt)_{\max } $$

where (*dx/dt*)_*max*_ is *the* maximum reaction rate of the sample, %/s, (*dx/dt*)_*mean*_ is the average reaction rate of the char samples, min^−1^, and *T*_max_ is the temperature corresponding to the maximum reaction rate, K, respectively. The calculated values of *S* and *R*_*w*_ are shown in Fig. 10, and the results shows that values of *S* and *R*_*w*_ decrease as the CG mixing ratio increased, which indicates that an increase in the CG mixing ratio is associated with a reduction in the combustion reactivity of the co-pyrolytic samples and an increase in the instability of the combustion process.

As known, the char combustion process involves a sequence of adsorption and desorption processes, which can be expressed as follows^45^:

$$ 2C_{f} + {\text{O}}_{2} \to 2{\text{C}}({\text{O}}) $$

$$ C_{f} + {\text{CO}}_{{2}} \to {\text{CO}} + {\text{C}}({\text{O}}) $$

$$ {\text{C}}\left( {\text{O}} \right) \to {\text{CO}} $$

$$ {\text{C}}\left( {\text{O}} \right) + {\text{O}}_{2} \to {\text{CO}}_{2} /{\text{CO}} + {\text{C}}({\text{O}}) $$

$$ 2{\text{C}}\left( {\text{O}} \right) \to {\text{CO}}_{2} + C_{f} $$

where *C*_*f*_ and C(O) are referred to an active/available site and a carbon–oxygen complex, respectively. The char combustibility is contingent upon its surface-active sites and porous structure. The pore structure and SEM results have demonstrated that the pore structure undergoes a fluctuating trend as the CG mixing ratio increases. The formation of large pores facilitates the contact between gas and active/available sites, which is conducive to combustion. This indicates that the change in pore structure has a minimal impact on the combustibility of the co-pyrolytic char. The results of XRD, FTIR and Raman Spectroscopy have demonstrated that the contents of oxygen-containing and aliphatic groups in the co-pyrolytic char samples decreased with the increase of the CG mixing ratio, whereas the contents of aromatic groups exhibited an inverse trend. Furthermore, the combustibility of oxygen-containing and aliphatic groups was found to be higher than that of aromatic groups^4^. This suggests that the reduction in the combustibility of the co-pyrolytic char samples with the increasing CG mixing ratio is primarily influenced by the chemical structure. Furthermore, CG will introduce quartz and mullite to react with the alkali metal in RS to form aluminosilicate (shown in Fig. 8), which will result in the alkali metal losing its catalytic activity, thereby reducing the combustibility of the co-pyrolytic char.

In order to further quantify the effect of the CG mixing ratio on the combustibility of the co-pyrolytic char, the kinetics of RS char and 80RS20CG were analyzed by the Kissinger–Akahira–Sunose (KAS) method^46^, and the equation was given as follow:

12 $$ \ln \left( {\frac{\beta }{{T^{2} }}} \right) = \ln \left( {\frac{{k_{0} R}}{EG(x)}} \right) - \frac{E}{RT} $$

where *G*(*x*) is the reaction mechanism function;* k*_0_ is the pre-exponential factor, min^−1^; *β* is the heating rate, K/min; *T* is the absolute temperature, K; *E* is the activation energy, kJ/mol; *R* is the gas constant (8.314 J/(mol·K)).

**Figure 11:**
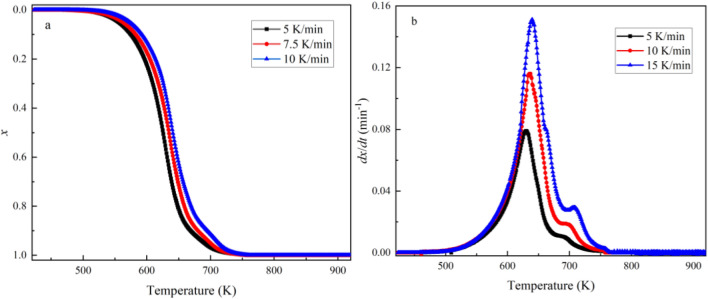
Combustion conversion rate (*x*) and reaction rate (*dx*/*dt*) curves of the RS char at different heating rates.

**Figure 12:**
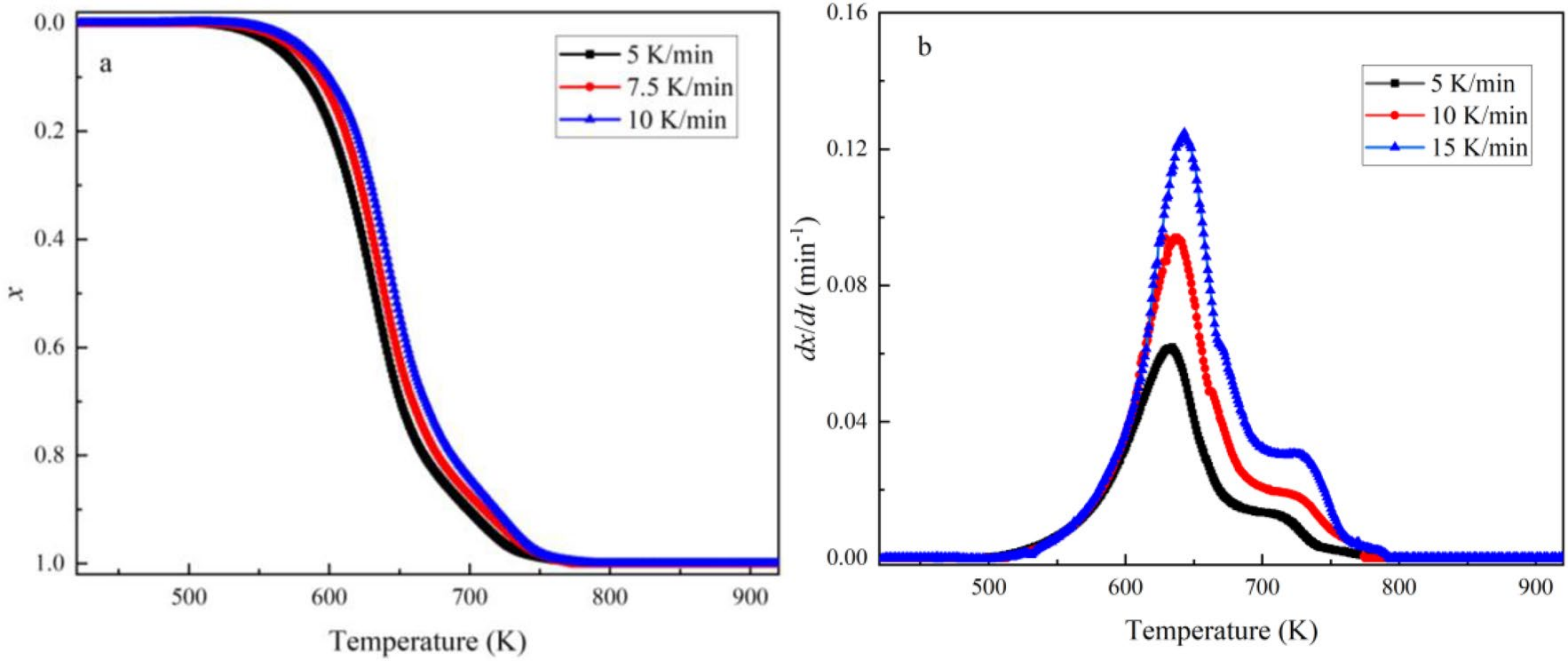
Combustion conversion rate (*x*) and reaction rate (*dx*/*dt*) curves of the 80RS20CG at different heating rates.

The curves of the combustion conversion rate and reaction rate for the RS char and 80RS20CG at different heating ratios under the air atmosphere are shown in Figs. 11, and 12. The results show that the combustion conversion rate curves shift to a higher temperature region as the heating rate increases, due to the time lag caused by the heat transfer at a higher heating rate, and the temperature rises to the higher level before the combustion has progressed sufficiently, resulting in a higher temperature range for char to undergo mass loss. In contrast, at a lower heating rate, the temperature distribution in the char particles becomes more homogeneous, resulting in more adequate combustion and less mass loss of the co-pyrolytic char sample.

**Figure 13:**
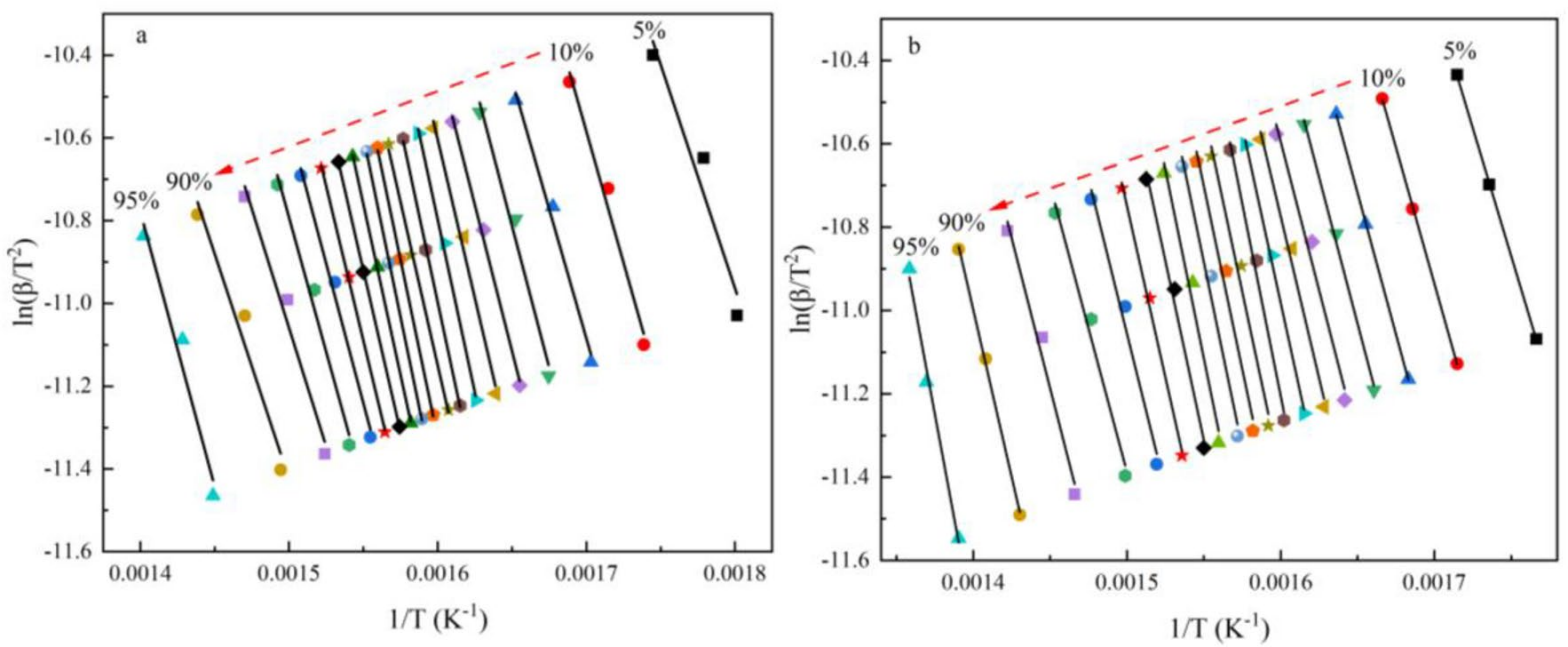
Kinetic fitting curves of the combustion conversion rate (**a**. RS char, **b**. 80RS20CG).

Whereas the KAS model adheres to a linear functional form (*Y* = *kX* + *b*) and the *E* could be determined by the calculated slope of the line plotting the left side of the equation against − 1/*T* for a given degree of conversion. In addition, the combustion of the co-pyrolytic char is generally considered to be a first order reaction, so the reaction mechanism can be described as *G*(*x*) = − ln(1 − *x*)^45,47^. Thus, the values of *k*_0_ can be calculated from the intercept of the fitted lines. The fitting curves are shown in Fig. 13, and the kinetic equations, values of *E*, *k*_0_ and correlation coefficients (R^2^) at different combustion conversion rates are presented in Table 5. As shown in Fig. 13 and Table 5, the correlation coefficients (R^2^) for each equation are consistently above 0.95, providing substantial confidence in the calculations and data reliability. The calculated *E* values of RS char at different conversion rates are in the range of 89.87–144.67 kJ/mol, which are lower than that of 80RS20CG (102.22–164.99 kJ/mol).

**Table 5:** Kinetic parameters of RS char and 80RS20CG at different combustion conversion rate.

Samples	*x*	Kinetic equation	*E* (kJ/mol)	*k*_0_ (min^−1^)	R^2^
RS char	0.05	y = − 10,809.82X + 8.49	89.87	2.63E+04	0.95
	0.10	y = − 12,675.23X + 10.96	105.38	7.30E+05	0.98
	0.15	y = − 12,501.07X + 10.17	103.93	4.90E+05	0.99
	0.20	y = − 13,693.99X + 11.78	113.85	3.58E+06	0.98
	0.25	y = − 14,111.97X + 12.17	117.33	6.81E+06	0.99
	0.30	y = − 15,325.94X + 13.92	127.42	5.11E+07	0.99
	0.35	y = − 16,710.41X + 15.94	138.93	4.90E+08	0.99
	0.40	y = − 16,838.52X + 15.95	139.99	5.70E+08	0.99
	0.45	y = − 15,805.32X + 14.14	131.41	9.86E+07	0.99
	0.50	y = − 17,231.20X + 16.24	143.26	9.76E+08	0.99
	0.55	y = − 17,400.88X + 16.37	144.67	1.23E+09	0.99
	0.60	y = − 16,496.65X + 14.81	137.15	2.68E+08	0.99
	0.65	y = − 15,581.89X + 13.23	129.55	5.66E+07	0.99
	0.70	y = − 14,908.54X + 12.02	123.95	1.74E+07	0.99
	0.75	y = − 13,487.15X + 9.66	112.13	1.59E+06	0.99
	0.80	y = − 13,009.17X + 8.72	108.16	6.40E+05	0.98
	0.85	y = − 11,486.69X + 6.17	95.50	4.69E+04	0.98
	0.90	y = − 10,866.92X + 4.88	90.35	1.29E+04	0.96
	0.95	y = − 13,269.38X + 7.80	110.32	3.09E+05	0.97
80RS20CG	0.05	y = − 12,295.26X + 10.65	102.22	2.66E+07	0.99
	0.10	y = − 13,068.73X + 11.28	108.65	1.09E+08	0.99
	0.15	y = − 13,582.1X + 11.69	112.92	2.63E+08	0.99
	0.20	y = − 14,015.76X + 12.10	116.53	5.63E+08	0.99
	0.25	y = − 14,266.01X + 12.23	118.61	8.41E+08	0.96
	0.30	y = − 15,532.96X + 14.08	129.14	7.22E+09	0.98
	0.35	y = − 16,934.11X + 16.11	140.79	7.24E+10	0.99
	0.40	y = − 18,469.68X + 18.35	153.56	8.79E+11	0.98
	0.45	y = − 17,320.53X + 16.33	144.00	1.28E+11	0.96
	0.50	y = − 17,547.18X + 16.50	145.89	1.78E+11	0.96
	0.55	y = − 17,775.08X + 16.67	147.78	2.46E+11	0.96
	0.60	y = − 18,062.66X + 16.88	150.17	3.55E+11	0.96
	0.65	y = − 17,184.25X + 15.32	142.87	8.12E+10	0.98
	0.70	y = − 16,461.94X + 13.94	136.86	2.24E+10	0.99
	0.75	y = − 14,880.22X + 11.26	123.71	1.60E+09	0.97
	0.80	y = − 13,765.71X + 9.26	114.45	2.33E+08	0.97
	0.85	y = − 14,412.22X + 9.71	119.82	4.51E+08	0.97
	0.90	y = − 16,044.56X + 11.46	133.39	3.50E+09	1.00
	0.95	y = − 19,844.85X + 16.04	164.99	5.50E+11	0.99

The structural characterization of co-pyrolytic char samples with varying mixing ratios of RS and CG demonstrate that the mixing ratio exerts a significant influence on the physicochemical properties of co-pyrolytic char samples, including their morphology, organic functional groups, and inorganic minerals. An increase in the CG mixing ratio will result in an increase in the aromatic, accompanied by a reduction in the content of aliphatic hydrocarbons and oxygen-containing groups. The presence of mullite and quartz in the CG will result in the formation of stable aluminosilicates when in contact with alkali metals in the RS, lead to the loss of the catalytic effect of alkali metals on the subsequent combustion of the co-pyrolytic char. While the pore structure shows an initial increase and subsequent decrease as the CG mixing ratio increased. The results of the combustion characteristics analysis indicate that the combustibility of the co-pyrolytic char decreases with an increase in the CG mixing ratio. The experimental findings and results presented above indicate that the aromatization of the carbon structure is a significant factor contributing to the reduced combustibility of the co-pyrolytic char.

A collaborative approach utilizing XRD, Raman, FTIR and TGA has systematically presented the influence of the mixing ratio on the evolution of the physicochemical properties as well as the combustibility of the co-pyrolytic char. This helps to elucidate the influence of the mixing ratio on the co-combustion behavior of RS and CG in a more comprehensive manner.

## Conclusion

The physicochemical properties and combustion characteristics of the co-pyrolytic char prepared under a N_2_ atmosphere with different mixing ratios of RS and CG were investigated in this study. The main conclusions are summarized as follows.

1. The introduction of CG will increase the ignition and burnout temperature, as well as prolong the co-pyrolytic char combustion time.
2. The deterioration in the combustibility of co-pyrolytic char with an increase in the CG mixing ratio can be attributed to the increase in the content of aromatic and the decrease in the content of aliphatic hydrocarbons and oxygen-containing groups.
3. The mullite and quartz in the CG will react with alkali metals in the RS to form aluminosilicate, which reduces the catalytic ability of alkali metals for the co-pyrolytic char combustion.
4. The combustion kinetics of RS char and 80RS20CG were analyzed by the KAS method, and the activation energy of the 80RS20CG is 102.22–164.99 kJ/mol, which is higher than 89.87–144.67 kJ/mol of RS char.

Future studies will extend to the investigation of feedstock types and operating conditions, including temperature and pressure, as well as pollutant emissions during the co-combustion of RS and CG.

## Data Availability

The datasets used and/or analyzed during the current study available from the corresponding author on reasonable request.
